# “Using Crowd-Sourced Data to Explore Police-Related-Deaths in the United States (2000–2017): The Case of Fatal Encounters”

**DOI:** 10.5334/ohd.30

**Published:** 2019-05-07

**Authors:** Brian Karl Finch, Audrey Beck, D. Brian Burghart, Richard Johnson, David Klinger, Kyla Thomas

**Affiliations:** 1 USC, US; 2 SDSU, US; 3 Dolan Consulting, US; 4 U. Missouri-St. Louis, US

**Keywords:** police-related-deaths, police homicides

## Abstract

**Objectives:**

We evaluated the Fatal Encounters (FE) database as an open-source surveillance system for tracking police-related deaths (PRDs).

**Methods:**

We compared the coverage of FE data to several known government sources of police-related deaths and police homicide data. We also replicated incident selection from a recent review of the National Violent Death Reporting System.

**Results:**

FE collected data on *n = 23,578* PRDs from 2000–2017. A pilot study and ongoing data integration suggest greater coverage than extant data sets. Advantages of the FE data include circumstance of death specificity, incident geo-locations, identification of involved police-agencies, and near immediate availability of data. Disadvantages include a high rate of missingness for decedent race/ethnicity, potentially higher rates of missing incidents in older data, and the exclusion of more comprehensive police use-of-force and nonlethal use-of-force data—a critique applicable to all extant data sets.

**Conclusions:**

FE is the largest collection of PRDs in the United States and remains as the most likely source for historical trend comparisons and police-department level analyses of the causes of PRDs.

## Introduction

Citizen deaths that occur during interactions with police officers are increasingly viewed by members of the general public and scholars as a public health concern in the United States [1, 2]. Although the term “police homicides” is often used in discussions of citizen deaths during police activities, we prefer the term “police-related deaths” (PRDs) as it permits definitional granularity within a broader net of police violence. PRDs include, among other things: police homicides (law enforcement officers killing citizens, justifiably or otherwise), citizens who die in automobile accidents during vehicle pursuits, citizens who suffer medical emergencies during interactions with police, and citizens committing suicide with police on-scene.

In a recent commentary in the New England Journal of Medicine, Crosby and Lyons argue that *legal intervention deaths* “are not only devastating to the victims’ families and the directly affected communities or neighborhoods; … they represent a significant public health burden and can incite further violence in which more people are killed” [2]. Crosby and Lyons call for a study of police homicides that systematically assesses the scope and nature of such deaths [2]. A related commentary notes that citizens being killed by the police “affect[s] the well-being of the families and communities of the deceased” [1]. Research also suggests that, beyond their impact on victims, families, and communities, police homicides can have long-lasting physical and mental health consequences for police officers, many of whom experience symptoms of “post-shooting stress disorder” [3–5].

An in-depth understanding of the broader community impact of police homicides and other PRDs requires thorough knowledge of the scope and nature of such deaths. Unfortunately, however, we lack reliable and comprehensive data about these sorts of deaths and the circumstances surrounding them. No public surveillance system in the United States counts PRDs and the government data collection efforts intended to capture some aspect(s) of the PRD phenomenon—for example, a) the Federal Bureau of Investigation’s Supplementary Homicide Reports, b) the Bureau of Justice Statistics’ Arrest-Related Deaths Reports, and c) the Centers for Disease Control’s National Violent Death Reporting System—are inconsistent and unreliable [6–8]. In recent years, however, some citizens have responded to the omissions and flaws in these official government-produced sources by developing data sets designed to produce more accurate and complete counts of citizens who die during interactions with police officers.

These unofficial data rely on internet crowd-sourcing and other data collection efforts conducted by the public to catalogue some aspect(s) of PRD’s; several researchers have suggested that these efforts may capture more citizen deaths [9] and may therefore be the best current strategy for collecting data on PRD’s [10]. Unfortunately, very little is known about the quality of the information contained in data sets produced by citizen researchers. The primary aim of this paper is to both summarize the state of official data sources and to further our understanding of unofficial data collections by analyzing the relative advantages, limitations, and completeness of one of the most prominent sources of PRD data assembled by citizens to date: the Fatal Encounters Project (http://www.fatalencounters.org/).

## Extant Data Sources

Currently, government-funded criminal justice data collections are comprised of two sources: 1) the voluntary justifiable homicides (JH) portion of the Supplementary Homicide Reports (SHR) collected under the Uniform Crime Reporting System of the FBI, and 2) the piecemeal Department of Justice’s arrest-related-death (ARD) data that is part of the Deaths in Custody Reporting Program (DCRP). Researchers have long known that these data contain substantial omissions [7, 11–12] and an internal review by the Bureau of Justice Statistics (BJS) notes that a majority of incidents may be missing from the arrest-related-death data [13], which have not been reported publicly since 2009. Currently, data from only 750 of approximately 17,985 (4.2%) law enforcement agencies voluntarily submit “justifiable homicides” to the FBI’s SHR program [6–7], and the BJS reports that between 31–41% of ARD in 2011 were not captured, with approximately 50% uncaptured for years prior (2003–2009) [14].

## Federally Mandated and Other Data Collection Efforts

The DCRP has been recently mandated to cover all law enforcement agencies [15] and is moving ahead with plans to collect pilot data; however, the ARD program relies on data submission from a single State Reporting Coordinator (SRC) from each state [16], rather reports mandated and produced by each police-department. Each SRC must collect his/her own data as law enforcement agencies are not required to systematically document or report incidents. These coordinators rely either exclusively (39%) or exclusively/partially (73%) on internet searches of news sources, while fewer than 20% use a law enforcement survey [14]. Going forward, we can expect this federally mandated program to continue to fail to capture a signification portion of PRDs—independent of the program being made mandatory for all states.

Given the clear liabilities of the DOJ data sources, some researchers have turned to other government data sources to measure police-related deaths, including: a) the National Vital Statistics Survey (NVSS), which is based on death certificates, and b) the National Violent Death Reporting System (NVDRS), which is based on death certificates characterized as having resulted from “legal intervention” [9, 17], as well as coroner/medical examiner and police reports.

In addition to the non-DOJ government data sets, some scholars have turned to crowd-sourced, internet-based sets developed by citizens, such as Killed by Police, The Counted, The Washington Post, Mapping Police Violence, and Fatal Encounters. However, very little is known about their quality, completeness, or reliability as data sources for quantifying the scope and nature of police-related deaths. To better our understanding of the strengths and weaknesses of citizen-based data sets that catalogue PRDs, we examine one of the most prominent citizen-based data sources assembled to date: the Fatal Encounters Project.

## Unofficial PRD Data: Fatal Encounters

PRD data for FE are collected using three methods: 1) Freedom of Information Act (FOIA) and other public records requests of law enforcement agencies, 2) crowdsourcing internet searches by volunteers, paid researchers, and the curator of FE, and 3) cross-checking of data with newly developing online websites such as those by The Guardian and Washington Post. These deaths include police homicides, deaths that occur due to suicides in police presence, accidental deaths during foot-pursuit, and accidental and use-of-force deaths during vehicular pursuits. Fatal Encounters is essentially a “living document” that is curated daily and is fact-checked and corrected by the curator and crowd-sources towards the end of comprehensively documenting all PRDs in the United States.

Newspapers and online news stories have both shown to be excellent sources of injury surveillance and reporting for a variety of phenomenon [18–22]. Further, crowd-sourcing of online/print news stories has shown to be a valid method to comprehensively assess prevalence for social and behavioral research in public health [23]. Finally, crowd-sourcing is a relatively low-cost and efficient way to collect information on newsworthy events such as PRDs.

## Advantages of FE

Although, as noted above, there are several crowd-sourced data sources that collect information on police homicides, Fatal Encounters is known to be a much more extensive source of data on PRDs in general, and police homicides in particular [9, 24]. FE collects data as far back as 2000 and contains more variables than other data sources. First, Fatal Encounters collects an extensive array of police-related deaths with a diverse set of causes and circumstances (see Table 1). This gives researchers the flexibility to explore diverse characterizations of deaths that work best with their research agendas. FE documents 23,578 incidents of PRDs from 2000–2017 for the entire nation. As of the date of manuscript submission (April 2019), FE documented 1,826 PRDs in 2018, a slight increase from the year-end estimate of 1,803. In addition, 400 PRDs have been documented through 4 months of 2019.

While incidents that would almost always be defined as police homicides (see e.g., gunshots and bludgeoning) comprise a substantial proportion of these 23,578 police-related deaths—several PRDs include circumstances of death such as asphyxiation, that are unlikely to be reported in death certificates or official government sources. Further, although gunshots are the most prevalent circumstance of police-related deaths, Table 1 reveals that various other circumstances account for a non-trivial portion of PRDs and that vehicular pursuits are the second most prevalent. According to Table 1, suicides during an arrest are the third most prevalent police-related death in FE; while many of these are instances of individuals turning a gun on themselves during a pursuit, others reflect grayer areas such as decedents who intentionally burned their homes during standoffs or who drowned while evading arrest.

A second advantage of FE is that its collection of data back to 2000 allows for important trend analyses of police-related deaths. Third, and perhaps most importantly, FE identifies a death as *police-related*, based on reports and follow-up reports made by journalists. Thus, it is not subject to the biases and social pressures that may inform whether a death is adjudicated to be the result of police involvement in an official document like a death certificate. For example, deaths ruled accidental in autopsy reports, may in fact be police-related deaths, and autopsy declarations by forensic pathologists can be heavily influenced by police-provided information or biased law enforcement authorities.

Fourth, every incident of a PRD in FE is linked to an address that has been geo-coded. To date, 97% of the 23,578 incidents of police-related deaths in FE have been geo-coded to an exact latitude/longitude. A pilot assessment [24] of the quality of these geo-codes using 15 years of police-related deaths in New York City (*n* = 384) found that addresses could be described by four tiers of specificity: Tier 1) 90.3% of incidents could be identified by an exact street address or name/cross-street combination (predicted error of ±50 m); Tier 2) 1.3% identified by *hundreds blocks* (“800 block of Main Street”, e.g.; predicted error ±200 m); Tier 3) 7.5% roads (“I–84”, e.g.; predicted error ±1,000–100,000 m); Tier 4) 0.7% places (“College of Staten Island”, e.g.; predicted error 1000m); and Tier 5) 0% no address. Of course, since FE data are open-source, errors can be (and are) corrected as needed.

Fifth, nearly all PRDs in FE are accompanied by news stories, which allows for a careful micro-analysis of each incident. Sixth, unlike the government sources of police homicide data, which can take several years for public release, FE’s ongoing data collection efforts, duplicate checks, and data cleaning, lead to incidents being released within one week of the date of death, often within a day or two.

Seventh, variable availability in FE is much more extensive than other sources and includes the following variables: decedent’s full name, decedent’s age, decedent’s gender, decedent’s race/ethnicity, URL image of decedent, date of incident/death, location of death, zip code of death, GPS coordinates, agency involved, circumstances of death (gunshot, vehicle, stun-gun, bludgeoned with instrument, beaten, medical emergency, asphyxiated, domestic violence, stabbed, drug overdose, bean bag rounds, other), details of the death (e.g., routine arrest, suspicion of activity, weapon present, decedent shots fired), indicators of symptoms of mental illness in the victim (e.g., suicidal threats, law enforcement called by family to assist with mentally ill family member), judicial disposition (justified, excusable, criminal, pending investigation, and others), and links to relevant news articles.

Finally, FE are open-sourced-data; corrections and omissions can be submitted by the public and although the ultimate determination for what appears in the file is determined by the FE curator, transparency is the primary motivating force behind the data collection and reporting, allowing individual researchers to make their own determinations.

## Limitations of FE

An important limitation of FE is that there is no gold standard with which to compare the completeness of the incidents collected in FE. In fairness, this is true for every data source used to quantify police-related deaths. A pilot study assessing the completeness of the FE data involved making FOIA requests of a random sample of 328 law enforcement agencies in a sample of 11 states (CT, FL, MA, ME, MT, NH, NV, NY, OR, RI, SD) was recently undertaken in order to assess the comprehensiveness of FE data for 2000–2015 (Farman 2016). Responses were obtained from 246 (75%) of the sampled agencies and it was found that FE data are *fully complete for 9 of the states* sampled. Data were missing for only 1 incident in CT (92% complete) and 8 in FL (95%); these incident news-stories have been located and added to the data. This pilot study further noted that FE data contained a substantial number of incidents that were not reported by police departments through public records requests. Additionally, although FE is the only online project to collect data before 2012—the Killed by Police (KBP) database collects data for May 2013–2018—compared to KBP, FE was 99.1% complete while KBP was 91.4% complete compared to FE for the years of overlap examined (2013–2015) [24].

A second limitation of FE is that it is possible that the reporting of older incidents is less complete due to PRDs being less historically newsworthy and/or the deletion of old internet news stories; on the other hand, the FOIA pilot did not indicate that older incidents were more likely to be missing from FE. A third limitation, and this critique applies to virtually all sources of PRD data (c.f., Vice News non-fatal police shootings), is that incidents reported by FE are only fatal outcomes and do not reflect the full continuum of police-related violence, gunshots that miss their target, and nonfatal gunshots and other uses of force that result in injury. Ultimately, researchers will need to document and collect this wide range of data in order to fully understand police use of force.

A fourth limitation with FE concerns missing data. While missingness is rare for most variables (e.g., name of decedent 2.7%, age 2.8%, gender 0.2%), nearly 40% of the cases (38.9%) are missing information about decedents” race/ethnicity. This is largely because this variable is coded based on news reports and accompanying photos in reported or related news stories. We implemented Bayesian-improved surname geo-coding [25] to replace missing race/ethnicity data. Using the non-missing incidents as a validation sample, we find that the use of surnames combined with Census demographic data at the level of the geo-coded incident block group yields statistically significant (p < .001) point bi-serial correlations with race/ethnicity as follows: non-Hispanic White, *r = .73*; non-Hispanic Black, *r = .72*; Hispanic, *r = .89*; Asian, *r = .73*; Native American, *r = .55*.

## Incident Count of FE Data

**Figure 1:**
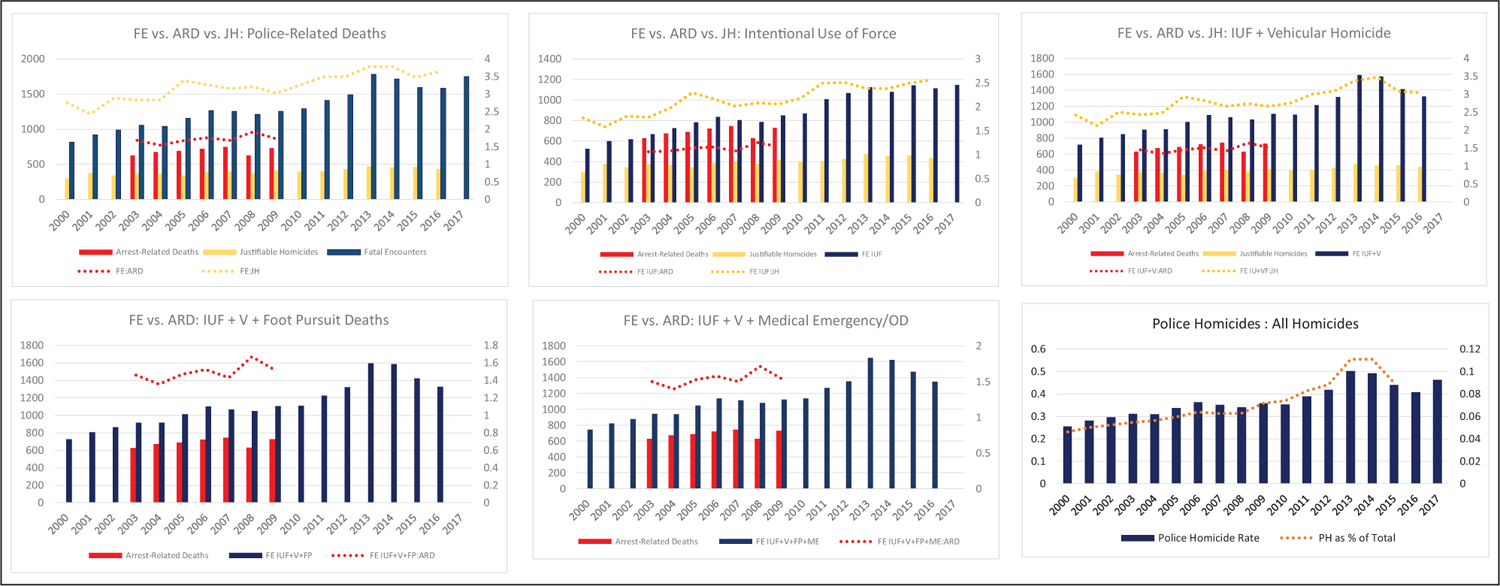
Fatal Encounters Officer-Related Deaths compared to DOJ Arrest-Related Deaths and FBI Justifiable Homicides.

To assess the coverage of incidents in FE, we first compared various circumstances of PRDs in FE to the DOJ’s arrest-related deaths (ARD) program (2000–2016) and the FBI’s justifiable homicides (JH) data (2003–2009). Although we include/exclude particular circumstances of deaths in each iteration, we use groupings that are most comparable for each data set comparison. For instance, panel 1 (the upper left panel) of Figure 1 compares all incidents of PRDs and finds that FE has as few as 1.55 times the number of incidents as ARD in 2004 and as many as 1.92 times the number of incidents in 2008. Compared to JH, FE has as few as 2.43 times the number of incidents in 2001 and as many as 3.78 times the number of incidents in 2013. Excluding suicides in police presence (see panel 5), FE still has as few as 1.39 times the number of incidents (2004) and as many as 1.72 times the number of incidents as ARD (2013). In fact, FE contains more incidents than both JH and ARD, no matter which types of PRD are counted, including: only intentional use of force (panel 2), intentional use of force plus vehicular homicides (panel 3), intentional use of force plus vehicular homicides and foot pursuit deaths (panel 4), and intentional use of force plus vehicular homicides, foot pursuit deaths, and medical emergencies and overdoses (panel 5). Finally, using a fairly restrictive definition of PRDs that include only intentional use of force and vehicular pursuit homicides (panel 6)—the number of police homicides as a percentage of the total number of population-based homicides has been steadily rising from a low of 5% in 2000, to a peak of 11.1% in 2013 and 2014, with a decline to 9.0% in 2015. At this time, lacking a true gold standard, there is no way to assess the completeness of the FE data, but we do note that FE contains substantially more incidents, even when definitions of incidents closely match. Tedious, but ongoing efforts are being made to ensure that incidents contained in all government data sets are also contained within FE.

Next, we replicated a strict criterion definition and selection for police homicides (2005–2012) that was detailed in a recent publication [9] that was found to be far superior to the NVSS and ARD data sources. Using these precise definitions, we found that overall, FE documented 10% more incidents of police homicide than the NVDRS (see Table 2).

## Discussion and Conclusions

The deaths in custody reporting program (DCRP) will be the only government mandated collection of police homicide data for the United States going forward. However, as noted, an internal review of this data collection system discovered large holes in coverage that were not simply reducible to voluntary data submission [13]. In addition, the FBI has recently begun a “National Use-of-Force Data Collection Program” (https://www.fbi.gov/services/cjis/ucr/use-of-force) which is designed to capture all shootings (missed shots, injuries, and deaths). This program began in January of 2019 and data are not yet available for assessment, but unfortunately, this program remains completely voluntary.

A mandatory FBI data collection would be much preferred to virtually all data collection efforts. Of course, as we have noted, this process—while collecting missed shots, injuries, and fatalities—would also exclude a large portion of police-related deaths due to non-firearm related violence such as car chase fatalities, asphyxiation, and taser deaths. As a result, the best option to date may be the collection of all police-related-death data, as is done in Fatal Encounters. This allows for crowd-sourcing incidents, makes the data open-source and usable by anyone, and eliminates error-prone or intentionally false decision-making as to which incidents to document. Preliminary analysis of FE notes minor disadvantages in the data set, but finds that FE contains the largest set of police-related-deaths in the United States for nearly two decades, indicating that it may be the most comprehensive, and may become more widely used [26, 27]. Trend analysis should be limited to more recent years, but the identification of responsible/involved agencies allows for police-department-level of analyses of PRDs, which could ultimately lead to policy-relevant changes in how police conduct their daily business, and ultimately contribute to decreasing the abhorrently high levels of violence and homicide in the United States.

## Figures and Tables

**Table 1:** Circumstance of Death by Year in Fatal Encounters.

Police-Related Deaths (PRD)	‘00	‘01	‘02	‘03	‘04	‘05	‘06	‘07	‘08	‘09	‘10	‘11	‘12	‘13	‘14	‘15	‘16	‘17	Total
**Intentional Use of Force (IUF)**																			
Asphyxiated/Restrained	19	16	22	9	10	12	19	8	5	9	13	7	12	12	3	8	16	15	215
Beaten/Bludgeoned	13	10	9	8	14	7	7	9	8	6	7	10	9	10	8	17	6	1	159
Burned/Smoke Inhalation		5	1		3	2		2				5	2	1	0	1	1	2	25
Chemical agent/Pepper spray	2			2	4	4	6	2	1	1	2		1		4	1		1	31
Gunshot	484	566	574	632	653	683	723	712	705	779	776	924	994	1058	1022	1057	1052	1090	14484
Stabbed	7	1		1	1	1	2		1	2	2	2		1	1			1	23
Tasered			11	14	41	74	79	69	67	54	66	60	51	43	39	60	40	35	803
**Vehicular Pursuit (V)**	193	204	234	240	182	216	252	257	248	251	228	206	245	465	495	272	206	358	4752
**Foot Pursuit (FP)**																			
Fell from a height	4	1	4	2		4	4	3	4	1	2	2	6	2	4	1	3	1	48
Drowned	7	4	10	8	6	9	7	6	11	2	12	10	3	4	10	8	3	3	123
**Medical Emergency (ME)**																			
Medical emergency	10	9	8	19	17	26	16	33	24	7	23	27	19	39	19	30	14	4	344
Drug overdose	4	4	4	5	7	10	19	13	5	10	5	15	10	12	15	18	5		161
**Suicide (S)**	70	97	101	89	96	101	119	131	125	126	147	130	133	132	84	113	229	238	2261
**Other/Unknown**																			
Other	1	1	2	22		1	3	2	1	1	0	2	2	2		2	4	1	47
Undetermined	3	4	6	6	7	3	7	8	6	6	5	14	2	4	8	8	4	1	102
Total	817	922	986	1057	1041	1153	1263	1255	1211	1255	1288	1414	1489	1785	1712	1596	1583	1751	23578

**Table 2:** Replication of Selection Criterion and Comparison with Barber et al. [9].

State	NVDRS No. (Rate)	NVDRS Abstractor No. (Ratio)	NVSS No. (Ratio)	SHR No. (Ratio)	FE No. (Ratio)
Alaska	24 (0.43)	26 (1.08)	10 (0.42)	0 (0.00)	25 (1.04)
Colorado	119 (0.30)	100 (0.84)	77 (0.65)	60 (0.50)	133 (1.12)
Georgia	228 (0.30)	184 (0.81)	108 (0.47)	92 (0.40)	279 (1.22)
Kentucky	66 (0.19)	45 (0.68)	37 (0.56)	19 (0.29)	76 (1.15)
Maryland	177 (0.39)	178 (1.01)	128 (0.72)	164 (0.93)	163 (0.92)
Massachusetts	46 (0.09)	46 (1.00)	36 (0.78)	6 (0.13)	52 (1.13)
New Jersey	95 (0.14)	95 (1.00)	31 (0.33)	82 (0.86)	121 (1.27)
New Mexico	82 (0.51)	78 (0.95)	66 (0.80)	41 (0.50)	87 (1.06)
North Carolina	166 (0.22)	161 (0.97)	33 (0.20)	43 (0.26)	133 (0.80)
Oklahoma	103 (0.35)	103 (1.00)	37 (0.36)	78 (0.76)	122 (1.18)
Oregon	91 (0.30)	95 (1.04)	94 (1.03)	29 (0.32)	99 (1.09)
Rhode Island	13 (0.15)	13 (1.00)		2 (0.15)	13 (1.00)
South Carolina	77 (0.21)	35 (0.45)	45 (0.58)	20 (0.26)	107 (1.39)
Utah	67 (0.31)	66 (0.99)	50 (0.75)	21 (0.31)	71 (1.06)
Virginia	119 (0.19)	118 (0.99)	104 (0.87)	39 (0.33)	133 (1.12)
Wisconsin	79 (0.17)	78 (0.99)	50 (0.63)	46 (0.58)	88 (1.11)
Total	1552 (0.24)	1421 (0.92)	906 (0.58)	742 (0.48)	1702 (1.10)
